# Author response to: Excision and suture in the midline *versus* Karydakis flap surgery for pilonidal sinus: randomized clinical trial

**DOI:** 10.1093/bjsopen/zrac106

**Published:** 2022-08-26

**Authors:** Oskar Hemmingsson, Felix Binnermark, Christoffer Odensten, Martin Rutegård, Karl A Franklin, Markku M Haapamäki

**Affiliations:** Department of Surgical and Perioperative Sciences, Surgery, Umeå University, Umeå, Sweden; Wallenberg Centre for Molecular Medicine, Umeå University, Umeå, Sweden; Department of Surgical and Perioperative Sciences, Surgery, Umeå University, Umeå, Sweden; Department of Surgical and Perioperative Sciences, Surgery, Umeå University Educational Unit at Sunderby Hospital, Sunderby, Sweden; Department of Surgical and Perioperative Sciences, Surgery, Umeå University, Umeå, Sweden; Wallenberg Centre for Molecular Medicine, Umeå University, Umeå, Sweden; Department of Surgical and Perioperative Sciences, Surgery, Umeå University, Umeå, Sweden; Department of Surgical and Perioperative Sciences, Surgery, Umeå University, Umeå, Sweden

*Dear Editor*

We welcome the comments written by Doll *et al*. who clearly appreciate the challenges in the treatment of pilonidal sinus disease.

We agree that existing knowledge should be incorporated in future study designs and we acknowledge the meta-analysis published by Stauffer *et al*. in 2018^1^ is likely the most ambitious analysis ever made. They reported that primary midline closure was associated with a 5-year recurrence rate of 16.8 per cent compared with 1.9 per cent after Karydakis surgery. They also reported that few randomized clinical trials (RCTs) existed and they lacked long-term follow-up data^1^.

In our study^2^, midline closure was used as the comparator as no RCT had compared this technique with the Karydakis flap surgery at the commencement of the study in 2006. The evidence of the Karydakis technique having superior outcomes compared with tension-free midline closure had not been confirmed and midline closure remained common in Sweden, despite previous recommendations against it^3^. Wound healing was expected to be faster after the Karydakis flap; however, previous data indicating this derive from observational studies and RCTs comparing Karydakis flap with other techniques other than midline tension-free closure. Our study provides long-term follow-up, as recommended by both Stauffer *et al*. and Allen-Mersh *et al*.^1,3^. An earlier RCT comparing the two techniques found no differences in wound complications or recurrence rate^4^. We found a median wound healing time of 49 days (95 per cent c.i. 32 to 66 days) after midline closure, whereas it was 14 days (95 per cent c.i. 12 to 20) days after Karydakis flap surgery.

Our study demonstrated shorter wound healing time after Karydakis flap surgery compared with midline closure. This supports previous reviews and adds to the evidence of superior outcomes using Karydakis flap surgery for pilonidal sinus disease compared with midline closure.
